# Mitochondrial Dysfunction Associated with mtDNA in Metabolic Syndrome and Obesity

**DOI:** 10.3390/ijms241512012

**Published:** 2023-07-27

**Authors:** Natalia Todosenko, Olga Khaziakhmatova, Vladimir Malashchenko, Kristina Yurova, Maria Bograya, Maria Beletskaya, Maria Vulf, Natalia Gazatova, Larisa Litvinova

**Affiliations:** 1 Center for Immunology and Cellular Biotechnology, Immanuel Kant Baltic Federal University, 236001 Kaliningrad, Russia; tod_89@mail.ru (N.T.); olga_khaziakhmatova@mail.ru (O.K.); vlmalashchenko@kantiana.ru (V.M.); kristina_kofanova@mail.ru (K.Y.); mbograya@mail.ru (M.B.); mariyabel@bk.ru (M.B.); mary-jean@yandex.ru (M.V.); n_gazatova@mail.ru (N.G.); 2 Laboratory of Cellular and Microfluidic Technologies, Siberian State Medical University, 634050 Tomsk, Russia

**Keywords:** metabolic syndrome, obesity, mitochondrial dysfunction, mtDNA, mitochondrial protease Lonp1

## Abstract

Metabolic syndrome (MetS) is a precursor to the major health diseases associated with high mortality in industrialized countries: cardiovascular disease and diabetes. An important component of the pathogenesis of the metabolic syndrome is mitochondrial dysfunction, which is associated with tissue hypoxia, disruption of mitochondrial integrity, increased production of reactive oxygen species, and a decrease in ATP, leading to a chronic inflammatory state that affects tissues and organ systems. The mitochondrial AAA + protease Lon (Lonp1) has a broad spectrum of activities. In addition to its classical function (degradation of misfolded or damaged proteins), enzymatic activity (proteolysis, chaperone activity, mitochondrial DNA (mtDNA)binding) has been demonstrated. At the same time, the spectrum of Lonp1 activity extends to the regulation of cellular processes inside mitochondria, as well as outside mitochondria (nuclear localization). This mitochondrial protease with enzymatic activity may be a promising molecular target for the development of targeted therapy for MetS and its components. The aim of this review is to elucidate the role of mtDNA in the pathogenesis of metabolic syndrome and its components as a key component of mitochondrial dysfunction and to describe the promising and little-studied AAA + LonP1 protease as a potential target in metabolic disorders.

## 1. Introduction

Obesity is the main factor in the development of metabolic syndrome (MetS), which is characterized by complications such as visceral (abdominal) obesity, hypertension, high cholesterol, and elevated glycemia (hyperglycemia). MetS is a precursor of major health diseases associated with high mortality in industrialized countries: cardiovascular disease and diabetes [1].

The World Health Organization estimates that the global incidence rate of metabolic syndrome in adults is over 25% and increasing every year. This increases the risk of developing type 2 diabetes (fivefold), having a heart attack (threefold), and dying (twofold) [2,3]. The increase in incidence is primarily related to the heterogeneous phenotypes of the MetS and the lack of specific effective therapies [3].

Adipose tissue and phagocytes are active producers of proinflammatory factors that contribute to the development of mild chronic inflammation associated with insulin resistance, type 2 diabetes mellitus, and MetS [4,5].

Chronic inflammation leads to accelerated biological aging of the body, characterized by a shortening of the length of cellular telomeres and the development of mitochondrial dysfunction [6,7].

At the same time, mitochondrial dysfunction, which is associated with tissue hypoxia, impaired mitochondrial integrity, increased production of reactive oxygen species, and a decrease in ATP, leading to a chronic inflammatory state that affects tissues and organ systems, is an important component of the pathogenesis of the MetS.

Mitochondrial dysfunction and cellular aging are also associated with a decrease in mitochondrial DNA (mtDNA) copy number [6] and an accumulation of mitochondrial mutations (heteroplasmy) [7]. At the same time, a positive correlation was found between telomere length and mtDNA copy number [4]. Studies have shown an association between MetS and its components with a decrease in mtDNA in peripheral blood cells [7,8]. In addition, low mtDNA levels have been found to be associated with various diseases and mortality [6].

The mitochondrial AAA + protease Lon (Lonp1) has a broad spectrum of activities. In addition to its classical function (degradation of misfolded or damaged proteins), enzymatic activity (proteolysis, chaperone activity, mtDNA binding) has been demonstrated. At the same time, the spectrum of Lonp1 activity extends to the regulation of cellular processes inside mitochondria, as well as outside mitochondria (nuclear localization). Lonp1 is ubiquitously expressed in humans and is involved in the regulation of mitophagy and response to oxidative stress, heat shock, and in the maintenance of mtDNA [8,9,10]. This mitochondrial protease with enzymatic activity may be a promising molecular target for the development of targeted therapy for MetS and its components.

The aim of this review is to elucidate the role of mtDNA in the pathogenesis of metabolic syndrome and its components as a key component of mitochondrial dysfunction and to describe the promising and little-studied AAA+ LonP1 protease as a potential target (with possible therapeutic potential) in metabolic disorders.

## 2. mtDNA

Each cell contains many mitochondria, and each mitochondria contains several copies of mtDNA [11]. mtDNA is located in the mitochondrial matrix in close proximity to the electron transport chain, the major source of reactive oxygen species. Therefore, mtDNA is particularly susceptible to oxidation leading to mutations and the development of various pathologies [12]. Human mtDNA is 16,569 bp in size and contains 37 genes encoding 22 transfer RNAs, two ribosomal RNAs required for protein synthesis, and 13 messenger RNAs required for OXPHOS [13].

The double-stranded closed mtDNA molecule consists of a heavy outer ring (H) and a complementary light inner ring (L) that contain no introns [14]. At the same time, the D-loop (control region) is the most intense regulatory non-coding region of mtDNA, which contains the origin of replication of the H-strand. It is noted that the mitochondrial control region is subject to a high rate of mtDNA changes (especially in hypervariable regions) [13].

Despite multiple repair pathways, cells have limited ability to repair mtDNA [12].

### 2.1. The Relationship between Quantitative mtDNA Levels (and Inflammation) in Obesity and Metabolic Syndrome

Studies show that each mitochondrion contains 2 to 10 copies of mtDNA (mtDNA CN) [11].

The mechanism underlying mtDNA CN alteration in metabolic disorders is directly related to excess energy substrates leading to insulin resistance mediated by an increase in inflammatory cytokine profile, resulting in mitochondrial fragmentation, oxidative stress (increased ROS production), accumulation of mtDNA damage, and cellular senescence [1]. Notably, there is evidence for a causal relationship between changes in mtDNA CN and inflammation in adipose tissue and oxidative stress. In adipose tissue, a decrease in mtDNA CN can lead to a deficiency in OXPHOS proteins and promote the formation of a pool of M1 macrophages that generate ATP through glycolysis and produce pro-inflammatory mediators, contributing to insulin resistance [15,16].

The tissue-specific indicator of the amount of mtDNA CN per cell is not a direct marker of mitochondrial function but is related to mitochondrial enzyme activity and adenosine triphosphate production [17]. This confirms the results of a study in which cells with reduced mtDNA CN had reduced expression of vital complex proteins, altered cell morphology, and lower respiratory enzyme activity [18].

Mitochondrial dysfunction has been shown to be associated with changes in the quantitative content of mtDNA and the degree of mtDNA damage [19] at MetS, insulin resistance, and obesity [20,21].

Studies in humans and animals have shown that loss of mtDNA content occurs in metabolic diseases [22]. The change in mtDNA CN in obesity has been described in studies that analyzed this parameter in adipose tissue [23] and peripheral blood [9].

Studies confirm a dynamic relationship between mtDNA CN and weight changes [24,25]. At the same time, a cross-sectional analysis of large cohorts showed a direct association between reduced mtDNA CN and obesity, diabetes, dyslipidemia, and hypertension [26].

A decrease in mtDNA CN in leukocytes, skeletal muscle cells, hepatocytes, and white adipocytes correlates with visceral obesity, body mass index, hyperlipidemia, cardiovascular disease, MetS, and mortality [5,27,28]. The results of studies with a limited sample of subjects diagnosed at MetS showed depletion of mtDNA CN in peripheral blood leukocyte cells [29,30].

In addition, lower levels of mtDNA CN correlated with MetS components in the general population [1,29,30].

In a recent study of a large cohort of patients with MetS and type 2 diabetes (type 2 DM), a strong association was found between low whole blood mtDNA CN levels and obesity, MetS, and type 2 DM. Moreover, mtDNA CN may predict the risk of developing MetS in patients with chronic kidney disease and in the European population as a whole [31].

Interestingly, a 10-year study in patients with MetS found an annual decrease of about five copies of mtDNA (in peripheral blood cells) [1], consistent with similar studies [6,32]. These studies draw a parallel between metabolic disorders and aging, as similar changes (decrease) in mtDNA copies per cell with age are observed in peripheral blood leukocytes [6], human pancreatic cells [33], fibroblasts, and myocytes.

In addition, higher triglyceride levels have been found to be associated with a decrease in mtDNA in peripheral blood mononuclear cells in patients with MetS [1], and similar associations have been shown for hyperglycemia and mtDNA [34].

A low baseline mtDNA CN was prospectively associated with a higher risk of developing type 2 DM [35]. It is noted that mtDNA CN level is a dynamic indicator and may change depending on endogenous/exogenous conditions. Thus, a higher mtDNA CN level was observed in type 2 diabetic patients with greater weight loss while taking the antidiabetic drug (metformin), which was associated with a decrease in glucose level, allowing the use of mtDNA CN for effective weight control in type 2 DM [36]. In addition, sex-specific variation in mtDNA CN was noted, as were the dynamics of this indicator before and after bariatric surgery [37]. In contrast, a decrease in mtDNA CN in peripheral blood was associated with an increase in mtDNA copy number in subcutaneous adipose tissue and TNF-a production in people with a high body mass index [23].

Thus, the above data suggest a direct link between mtDNA CN alterations and the development of metabolic disorders such as obesity, type 2 DM, and MetS and confirm the important role of mitochondrial dysfunction in the development of these diseases.

### 2.2. mtDNA Methylation

A close relationship has been established between mitochondria/mtDNA and nuclear DNA.

The mtDNA is essential for normal mitochondrial function, and many mitochondrial proteins are encoded by nuclear genes [38]. The interaction between mitochondria and the nuclear epigenome is bidirectional: mitochondria mediate epigenetic processes, and changes in the epigenome control mitochondrial function and influence stress response and lifespan [39,40].

Mitochondria provide for the formation of intermediate metabolites necessary for epigenetic modifications in the nucleus that control mitochondrial protein expression.

Interestingly, mtDNA CN also regulates nuclear gene expression through changes in nuclear CpG methylation, which leads to more severe pathology by regulating signaling pathways [41]. Epigenetic changes in nuclear DNA and low levels of mtDNA CN have been found to correlate with lower cancer survival [41].

This is of particular interest because the measurement of DNA methylation in peripheral blood cells can be used as a marker of less accessible tissues involved in obesity [42], and differences in peripheral blood cell DNA methylation profiles have been demonstrated in obesity [43].

The phenomenon of mtDNA methylation was discovered in the 1970s. CpG and non-CpG methylations were detected in mtDNA, and the presence of methyltransferases in mitochondria was also demonstrated. At the same time, in the human mitochondrial genome, the number of C sites (subject to methylation) of the L chain is more than twice the number of C sites of the H chain, with a small number of the C sites being dinucleotide CpG. Thus, methylation of mtDNA occurs mainly at the L chain and CpG sites. Moreover, 12 of 13 protein-coding genes use the L-chain as a template. L-chain methylation could affect mitochondrial function by controlling mtDNA gene expression [44].

The mechanism of methylation due to mtDNA changes suggests the involvement of histones. Thus, retrograde signaling from mitochondria to the nucleus regulates histone acetylation and alters gene expression in the nucleus through heterogeneous ribonucleoprotein A2 (hnRNAP2). At the same time, histone modifications correlate with mitochondrial content and are associated with chromatin activation (H4K16, H3L4me3, H3K36me2) [45]. Moreover, oxidative phosphorylation injury alters methylation processes due to modifications of the methionine cycle involved in the formation of S-adenosylmethionine, a methyl donor for histones and DNA methyltransferases [41].

Mutations and single nucleotide polymorphisms occur more frequently in the mitochondrial displacement loop (D-loop) than in other mtDNA regions [46]. This is mediated by the high sensitivity of the D-loop of mtDNA to ROS-dependent damage.

Functional regulation of mtDNA in metabolic disorders has been shown to be related to methylation marks in mtDNA (particularly in the bias loop, D-loop) [39].

Oxidative stress leads to increased expression of a key transcription factor that controls mitochondrial gene expression and mtDNA replication, mitochondrial transcription factor A (TFAM). TFAM initiates mitochondrial transcription by binding to the D-loop region and also regulates the process of mtDNA replication.

At the same time, D-loop methylation of mtDNA also affects mitochondrial gene expression and mtDNA replication [47]. High D-loop methylation disrupts TFAM, thereby reducing mitochondrial gene transcription and altering mitochondrial function [48,49]. A change in mtDNA copy number was found in response to an increase/decrease in the level of D-loop methylation [50,51]. Hypomethylation of the D-loop enhances gene expression [49] and leads to an increase in mtDNA copy number. Hypermethylation was associated with a decrease in mtDNA copy number [52].

According to the literature, mammalian mtDNA is enriched in N6-methyldeoxyadenosine (6 mA), presumably involving the methyltransferase METTL4, which causes suppression of mtDNA binding to TFAM. Hypoxia contributed to a 1300-fold increase in 6 mA labeling in mtDNA compared with total DNA. Furthermore, 6 mA mtDNA methylation promoted attenuation of mtDNA transcription, decreased mtDNA CN (HepG2 cells), and impaired mitochondrial activity [53].

A paper by Bordoni L. et al. [54] shows that mtDNA methylation patterns in DNA samples from buccal swabs are associated with body composition in overweight children, confirming previous studies on sex differences in mtDNA CN and obesity [5,37].

However, another paper by Bordoni L. et al. [55] found slight associations between mtDNA methylation levels and obesity, with no association between mtDNA methylation and mtDNA CN in VAT biopsies. At the same time, higher D-loop methylation was observed in severe obesity [55].

Studies show a direct association between changes in mtDNA methylation and the development of obesity [56] and cardiovascular disease [57].

High levels of mtDNA-D loop methylation in peripheral blood were found to be associated with low mtDNA-CN levels in insulin resistance and obesity [58].

Thus, mtDNA methylation may have a modulatory effect on the expression of important genes involved in the maintenance of mitochondrial and cellular function in the background of metabolic disorders.

### 2.3. mtDNA Mutations Accumulated during Development or in Postmitotic Tissues

The mitochondrial genome has a high mutation rate, about 100–1000 times higher than that of the nuclear genome. The mtDNA is not protected by histone proteins, so defective genes accumulate in it 10–20 times faster than in nuclear DNA [59].

Pathological mutations in mtDNA persist through mitochondrial fusion/fission processes and impaired mitophagy [60,61]. Accumulation of somatic mutations in mtDNA (heteroplasmy) leads to disruption of mitochondrial functions [62].

mtDNA mutations play an important role in the pathogenesis of MetS.

Mutations of tRNA genes are localized in areas important for tRNA stability, tRNA concentration in equilibrium in mitochondria, and aminoacylation activity, leading to high ROS production, development of oxidative stress, and apoptosis [59]. Mutations in the mitochondrial tRNA allele T4291C have been observed in the European population and have been associated with MetS and metabolic defects. In addition, mtDNA D-loop mutations, particularly T16189C, have been associated with insulin resistance and coronary heart disease [63,64]. Diabetes-associated mtDNA mutations: tRNALeu(UUR) A3243G heteroplasmy is involved in the pathogenesis of hereditary diabetes [65]. Studies on the role of the A3243G mutation have shown a direct link to mitochondrial dysfunction associated with inefficient aminoacetylation, impaired mRNA precursor processing, and basic posttranscriptional modification of tRNALeu(UUR) [66]. A study of the relationship of mitochondrial heteroplasmy in peripheral blood cells in a man with juvenile (as well as familial) diabetes, MetS, and a history of multiple sclerosis showed interesting results. Analysis of the mtDNA genome revealed a heteroplasmic mutation of mtDNA A8890G exclusively in the patient studied (but not in his relatives), whereas an association between the MetS and the single nucleotide polymorphism T16189C was found in all family members [67].

Thus, investigating the role of mtDNA polymorphisms in the pathogenesis of MetS and its components is a promising direction that will enable future work to prevent and predict the likelihood of developing severe pathologies and reduce the high risk of mortality (outcomes).

### 2.4. mtDNA Haplogroups

mtDNA variants are inherited maternally without recombination and can accumulate over time. A mitochondrial haplogroup groups together people who have the same accumulated mtDNA variants and may be geographically separated. In other words, haplogroups are ancient functional polymorphisms associated with specific mtDNA lineages [68]. Different haplogroups form different branches of the mitochondrial phylogenetic tree [13].

Studies have shown the association between mitochondrial haplogroups and the development of obesity [69,70,71] and MetS [72] in different parts of the world.

Studies have shown the association between Asian haplogroups (Japanese, Korean) and type 2 DM and MetS. Haplogroup N9a was associated with a low risk of type 2 DM and haplogroups D4/D5 and F with a high risk of type 2 DM in a cohort of Japanese and Koreans [73]. The Japanese also showed an association between haplogroup M8a (8684C˃T) (polymorphisms of single mitochondrial nucleotides) and high risk for type 2 DM and between B4c (3497C˃T, 1119T˃C) and obesity [74]. On the contrary, in the Chinese population, haplogroup N9a (N9a1, N9a10a) is predisposed to the development of type 2 DM [75]. Haplogroups M9 [76] and M8a [77] were markers of increased risk for type 2 DM in Han Chinese. A study in the Taiwanese population showed a protective role against the development of type 2 DM haplogroup D4 and a predisposing role in terms of B4a1a, E2b1 [78,79]. An association between a predisposition to type 2 DM and the presence of D4 H haplogroups was found in the Chinese Uighur population [80].

At the same time, the study by Chalkia D. et al. [72] showed the association between mitochondrial haplogroups and the risk of developing MetS.

Therefore, further studies on the relationship between mitochondrial haplogroups and the development of MetS will provide more clarity on the molecular features of the pathogenesis of the disease and allow the identification of specific targets to prevent the development of severe complications.

### 2.5. mtDNA Released from Cells as Alarmins and Inflammation Inducers

According to the “endosymbiont theory” proposed by Lynn Margulis, it is the evolutionary transformation of an autonomous endosymbiont bacterium into a specialized cell organelle, the mitochondrion, that results in mitochondrial products being potentially recognized by the immune system as “foreign” molecules [81]. The pro-inflammatory function of mtDNA was first demonstrated in 2004 [82]. Moreover, oxidation is an essential component of the mtDNA-associated inflammatory response [83,84]. When mtDNA binds to TFAM (mitochondrial transcription factor), the nucleotide is very stable; otherwise, mtDNA is in a fragile and easily degraded state. The study showed that cell-free mtDNA and TFAM-associated mtDNA play important roles in the inflammatory response [85].

Mitochondria-released mtDNA (which, like bacterial DNA, contains unmethylated CpG motifs) is a key factor in the activation of innate immunity mediated by pathogen-associated pattern recognition receptor (PRR) (PAMP) signaling cascades. These include the following signaling pathways: cGAS (cyclic GMP-AMP synthase pathway), Toll-like receptors (TLR9), NOD-like NLR receptors, the inflammasome NRLP3 (protein-3 with NACHT, LRR, and PYD domains) [86], and retinoic acid-inducible gene 1 (RIG-1)-like receptors (RLRs) and C-type lectin receptors (CLRs) [12,87,88,89].

Recurrent sterile inflammation (chronic production of pro-inflammatory cytokines), mediated in part by the release of mtDNA, is a classic example of a pathophysiological condition that occurs in patients with metabolic diseases [90]. It is interesting to note that in the elderly (over 90 years of age), plasma mtDNA levels gradually increase with age and correlate positively with plasma levels of TNF-a, IL-6, IL-1ra, RANTES [91]. Given the important pathogenic role of (high) circulating cytokines (TNF-a, IL-6, IL-1) and their association with poor disease prognosis [92], it is important to understand the mechanisms of association between mtDNA and the development of an inflammatory response in obesity and MetS.

Uncovering the role of mtDNA in MetS-associated inflammatory processes will therefore allow the identification of potential targets for early diagnosis and the prevention of severe disease sequelae.

### 2.6. Signaling Pathways Activated by mtDNA in Obesity and Metabolic Syndrome

#### 2.6.1. cGAS-STING Signaling Pathway

Interferon gene stimulator (STING) is an inflammatory endoplasmic reticulum (ER) adapter protein (encoded by *TMEM173* gene) that causes chronic inflammation [93]. The nucleic acid pattern recognition receptor (cyclic GMP-AMP synthase, cGAS) is the upstream protein that activates STING. It has been found that cGAS can be localized on the cell membrane, in the cytoplasm, and in the nucleus. cGAS binds to double-stranded DNA of exogenous and endogenous origin (including mtDNA) [94]. The binding of cGAS to mtDNA induces the synthesis of cyclic dinucleotide (CDN)-2′,3′-cyclic guanosine monophosphate-adenosine monophosphate (cGAMP). The synthesized cGAMP binds to the active pocket site STING and activates further signal transduction [95].

cGAS-dependent and -independent (ER stress) stimulation of STING leads to subsequent transport and activation of TANK-binding kinase 1 (TNK1) for phosphorylation of the regulatory factor IFN 3 (IRF3). IRF3 then dissociates and dimerizes and becomes transcriptionally active. IRF3 then migrates to the nucleus and triggers an IRF3-dependent IFN-1 response [96]. In addition, TNK1 induces nuclear transfer of nuclear factor kappa B (NF-kB), which triggers the transcription of pro-inflammatory cytokines [97].

In addition, the cGAS-STING-TBK1 pathway, which involves IKK kinases [98], promotes the translocation of nuclear factor-kappa B (NF-kB) to the nucleus [99], which also regulates the transcription of pro-inflammatory factors. STING has been shown to induce canonical/noncanonical activation of the NF-kB pathway. Thus, activation of STING (via cGAS) stimulates NF-kB and mitogen-activated protein kinase signaling via TNK1 [97].

In vitro/in vivo studies have shown that TNK1 is essential for STING-induced NF-kB activation and downstream STING kinases (TBK1, IkB kinase ε-IKKε) act redundantly to mediate stimulation of STING-NF-Kb, which triggers transcription of pro-inflammatory cytokines [97,100].

In addition to the inflammatory response, STING also regulates the process of autophagy/mitophagy, lytic cell death, apoptosis, etc. In this regard, these signaling outputs may or may not depend on upstream cGAS and second messenger cGAMP [101].

It was found that in the background of MetS there is a violation of mitophagy processes responsible for the removal of damaged mitochondria. In addition, metabolic diseases are associated with a decrease in mtDNA regulators and changes in mitochondrial integrity and structure. This contributes to mitochondrial stress and mtDNA release, leading to inflammatory responses associated with obesity and MetS [102].

Studies have shown that high levels of mtDNA fragments in plasma and mtDNA damage are positively correlated with chronic inflammation and oxidative stress [103,104]. This suggests an important role of free mtDNA as DAMPs and activators of the cGAS-STING-TBK1 signaling cascade.

TFAM heterogeneity was found to promote the release of mtDNA into the cytosol and maintain cGAS. Thus, mtDNA in the cytosol can activate the cGAS-STING pathway and increase the expression of interferon-stimulated genes (ISG) through IFN-1 signaling [105].

Studies of adipose tissue in an animal model (mouse) fed a high-fat diet (HFD) and in obese humans have shown high expression of the transcription factor IRF3, which is associated with inflammation, macrophage infiltration, and insulin resistance [106]. In addition, Bai’s research group [107] investigated the mechanism of activation of the cGAS-cGAMP-STING signaling pathway in obesity that leads to insulin resistance, sterile inflammation, and energy dysregulation. Adipose tissue shows overexpression of an oxidoreductase-like protein with a disulfide bond (DsbA-L). DsbA-L is a mitochondrial matrix protein that is also localized to the endoplasmic reticulum (ER) and has a chaperone function in adiponectin multimerization and is involved in the folding of key mitochondrial proteins critical for mtDNA replication [108]. Obesity suppresses the expression of DsbA-L and destabilizes mtDNA, promoting its release and activation of the cGAS-STING pathway, which increases the expression of *TBK1*, *NF-kB*, *P65*, and *IRF3* genes. Increased levels of TBK1, NF-kB, P65, and IRF3 suppress insulin signaling and promote the inflammatory response.

Moreover, phosphorylated TBK1 negatively regulates thermogenesis and energy expenditure by activating phosphodiesterase-PDE3B/PDE4 to suppress protein kinase A (PKA) signaling in adipocytes and HFD-treated mice [107,109]. Simultaneously, overexpression of DsbA-L protected against mtDNA-induced stimulation of the cGAS-STING pathway and suppressed inflammatory factors via a mechanism unrelated to adiponectin and ER localization. Overexpression of DsbA-L restored PKA signaling and increased energy expenditure [107,109]. Moreover, HFD-induced obesity activates TBK1 kinase and deregulates energy homeostasis by directly inhibiting AMPK at Thr142 of the AMPKα subunit in adipose tissue [110]. At the same time, TBK1 has bidirectional activity against the inflammatory response in adipocytes [107,110]. In this study, obesity was found to trigger the release of mtDNA and cause activation of a signaling pathway.

Therefore, a more detailed investigation of the molecular aspect of mtDNA-mediated activation and the cGAS-STING-IRF3 pathway in the development and progression of the MetS will allow the identification of specific targets for effective regulation of recovery processes and reduce the risk of developing severe pathologies.

#### 2.6.2. TLR9

Toll-like receptor 9 (TLR9) is an intracellular pattern recognition receptor and is expressed in immune cells (dendritic cells, B lymphocytes, NK cells, macrophages, etc.) and non-immune cells (cardiomyocytes, muscle, and endothelial cells) [98,111].

Mitochondrially released mtDNA interacts with TLR9 against a background of oxidative stress and decreased autophagy and promotes an inflammatory response (in mice) [112]. It was found that mtDNA is taken up by immune cells in which it triggers an inflammatory response by activating TLR9 [113]. Activation of TLR9 via unmethylated mtDNA-CpG motifs leads to the induction of NF-kB and MAPK signaling cascades [89,111].

Interestingly, mtDNA-bound TFAM can interact with the plasma membrane receptor RAGE and induce internalization of mtDNA, facilitating its recognition by TLR9 [98].

TLR9 is known to signal through myeloid differentiation protein 88 (MyD88), which activates kinases and transcription factors: MAPK, NF-kB, IRF7, to enhance proinflammatory processes and type I interferon response. Studies have shown that full-length TLR9 is translocated through the Golgi apparatus and endolysosomal compartment under the guidance of the helper protein UNC93B1 and then cleaved to acquire the ability to take up DNA [114]. The recruitment of TLR9 to the endolysosome is a key moment for the formation of its proteolytic activity [98].

In peripheral blood monocytes from patients with MetS, increased expression of endosomal TLR9 was found compared with controls, which correlated with increased nuclear expression of NF-kB [115].

In addition, work in Tlr9-/- mice showed a lower inflammatory response compared to wild-type mice fed a high-calorie diet and demonstrated the relationship between adipocyte degeneration, free DNA release, TLR9 activation, and the development of adipose tissue inflammation in an obesity background [116].

In a recent study, patients with MetS were found to have increased levels of oxidized mtDNA (ox-mtDNA) in plasma and increased TLR9 expression in PBMCs. At the same time, rats receiving HFD also had increased levels of ox-mtDNA expression in plasma and TLR9 expression in cardiac immune cells. Interestingly, in patients with MetS, an increase in circulating ox-mtDNA activated the TLR9-NF-kB pathway and stimulated secretion of IL-1β, IL-6, and IL-8 (a block of culture work on THP-1 cells) [117].

Thus, the broad spectrum of activity of the activated TLR9 pathway under the influence of mtDNA suggests its key role in the pathogenesis of MetS and may serve as a promising target whose regulation will reduce the negative consequences of the disease.

#### 2.6.3. Pyrin Domain of NLR Family-Containing Inflammasome 3 (NLRP3)

Inflammasomes fall into several categories: (1) nucleotide-binding domain (NOD); leucine-rich repeats (LRR), containing protein (NLR), or NLR inflammasomes; (2) absent in melanoma 2 (AIM2) and pyrin inflammasomes; (3) non-canonical inflammasomes [118]. mtDNA is recognized by two members of the immunogenic receptor superfamily: AIM2 and NRLP3. NRLP3 inflammasomes are cytoplasmic multiprotein complexes of the innate immune system [81]. NRLP3 inflammasomes are localized in neutrophils, monocytes, dendritic cells, macrophages, and nonhematopoietic cells [119].

The inflammasome is activated by PAMPs and DAMPs (including mtDNA). The inflammasome is activated by two sequential signals. First, NF-kB is stimulated (via TLR9, TLR4, TLR7), which triggers the inflammatory response and prepares the cell for further activation. The second signal is a trigger that interacts with the recognition part of the complex and leads to oligomerization/activation of the inflammasome particle. The specific inflammasome protein is the recognition protein and the common one is the scaffold adaptor protein ASC that relays the caspase-1 signal. Assembly and oligomerization of the inflammasome lead to cleavage of caspase-1 and trigger secretion of IL-1β, whose expression was triggered by the start signal [98].

When NRLP3 is activated, the caspase 1 subunit of the NRLP3 complex cleaves pro-interleukins into mature IL-1β, IL-18, key markers of mild inflammation. NRLP3 is considered a key factor responsible for the initiation and progression of chronic inflammation [5].

Obesity, diabetes, and MetS have been shown to be associated with increased NRLP3 activity in subcutaneous adipose tissue (SAT). At the same time, inflammatory mediators, IL-1β, IL-18, and caspase-1 levels at SAT are significantly increased in patients with MetS compared to controls, which may contribute to an increase in insulin resistance, inflammation, and SAT fibrosis [120].

In addition, disruption of NRLP3 in adipose tissue decreases levels of pro-inflammatory cytokines and restores insulin sensitivity in obese mice [121].

An interaction between mtDNA and NLRP3 has been described for autophagic protein-deficient mouse macrophages (LC3B, beclin1). Against the background of LC3B/beclin1 deficiency, accumulation of dysfunctional mitochondria and NLRP3/ROS-dependent mtDNA translocation to the cytosol were observed. Cytosolic mtDNA was involved in the development of the inflammatory response through the activation of IL-1β/IL-18 secretion [122]. Studies have also shown that autophagic clearance of damaged mitochondria (and mtDNA) prevents the development of inflammation. In addition, the compound 8-OH-dG in ox-mtDNA has been suggested to play an important role in NLRP3 activation [123].

**Figure 1:**
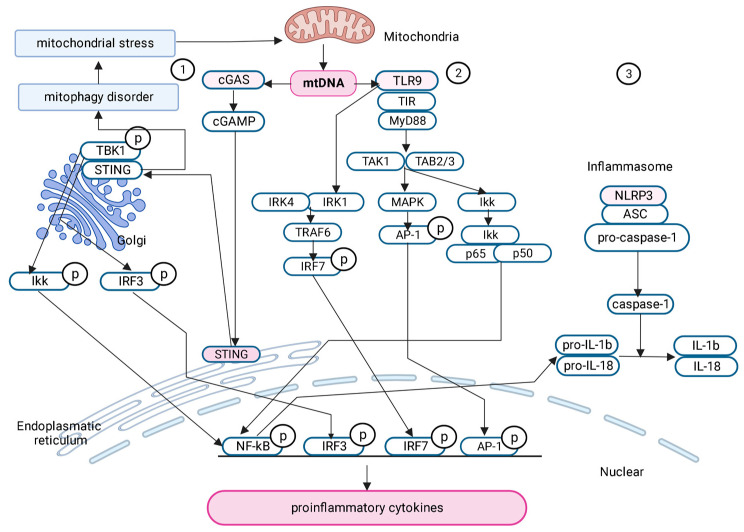
Signaling pathways activated by free mtDNA in the pathogenesis of metabolic syndrome. (1) cGAS- STING signaling pathway; (2) TLR9 signaling pathway; (3) NLRP3 signaling pathway.

Thus, MetS is directly related to the development of chronic, noninfectious (sterile) inflammation associated with mitochondrial damage and the release of mtDNA into the cytoplasm, which activates various signaling pathways (Figure 1).

Gradually, some aspects of the molecular action of mtDNA in the development of metabolic diseases are becoming better understood, but many questions remain unexplored and need to be investigated in depth.

## 3. Mitochondrial Protease Lonp1

The highly conserved ATP-dependent mitochondrial protease Lon AAA + (Lonp1) is a product of the nuclear *LONP1* gene, provides mitochondrial proteostasis (homeostasis, protein synthesis, folding, conformational maintenance, and degradation), and regulates adaptive cell responses to stress [124]. Studies have shown that LonP1 activation in vitro is associated with acute cellular stressors: hypoxia, oxidative stress, nutrient starvation, and unfolded protein response in the ER [125]. Lonp1 is an important regulator of mitochondrial metabolism and response to free radical damage, as well as an important factor in mtDNA maintenance and repair. The protease activity of Lonp1 is mediated by its circulation between binding (at the inner surface of the mitochondrial membrane) to the mitochondrial genome and release into the mitochondrial matrix, where it can cleave matrix proteins.

The functional potential of Lonp1 can be divided into three components:

- Proteolytic digestion of oxidized proteins and turnover of specific key mitochondrial enzymes: aconitase, TFAM, StAR (steroidogenic acute regulatory protein), P450, SP -22, COX4;
- Proteinchaperone interacting with the HSP60-mtHSP70 complex, GRP78, NDUF58;
- mtDNA-binding protein involved in mtDNA replication and mitogenesis [126].

Activation of LonP1 (in vitro) has been found to maintain normal cell viability in response to acute cellular stress [127], but in another study, opposite results linked LonP1 activation to cell death [128]. Overexpression of LonP1 has been observed during the oncogenic transformation of tumor cells, likely allowing overcoming hypoxic, metabolic, and proteotoxic stress [127,129]. However, the function of LonP1 in obesity and MetS in vivo is unknown.

Considering that the incidence of MetS is increasing every year and there are not enough potential therapeutic targets to address this important public health problem, investigating the role of LonP1 in obesity and MetS is an urgent task for the scientific community.

It has been found that homozygous deletion of the *LONP1* gene in mice results in early embryonic lethality, whereas heterozygous LONP1+/− mice are phenotypically indistinguishable from the norm [127]. The first described human disease mediated by pathogenic mutations in *LONP1* is a rare multisystemic developmental disorder affecting the brain, eyes, teeth, ears, and skeletal system in children, termed CODAS syndrome [130,131]. Although some patients have atrioventricular defects and incomplete cardiac septum [130].

In humans, LonP1 is ubiquitously expressed [8]. The highest expression of LonP1 is found in the heart, brain, liver, and skeletal muscle [132], indicating its important role in maintaining mitochondrial homeostasis in energy-demanding tissues [132].

Studies in rats have shown that LonP1 maintains mitochondrial redox status and reduces protein oxidative damage and cell apoptosis by selectively inhibiting nicotinamide nucleotide transhydrogenase (Nnt) complex I subunit activity [132]. At the same time, aging was found to be associated with a decrease in LonP1 expression, whereas overexpression increased lifespan [132].

Thus, LonP1 protease may play a key role in the pathogenesis of metabolic syndrome and indirectly regulate the magnitude of the inflammatory response, providing a potential target for the prevention of complications of the disease.

### 3.1. Molecular Functions of LonP1 in Obesity and Metabolic Syndrome

Mitochondrial homeostasis requires the induction of several signaling pathways responsible for mitochondrial quality control, including the mitochondrial Unfolded Protein Response (UPRmt). UPRmt detects proteolytic perturbations specifically in mitochondria and removes the stress by retrograde signaling to the nucleus, leading to transcriptional activation of protective genes [133]. Damaged proteins are recognized by mitochondrial chaperones (Hsp60) and members of the DnaJ heat shock protein family (Hsp40) and then unfold. This turns these proteins into substrates for proteases, including the LON protease [133,134,135,136], which degrades them [137].

LON protease (LONP1) is an important mitochondrial matrix enzyme that regulates the degradation of oxidized proteins and is strongly induced in response to acute stress [138].

The effect of UPRmt signaling components on metabolic phenotype has been demonstrated [139]. Inhibition of the OXPHOS complex in skeletal muscle and adipose tissue activates UPRmt, which plays a protective role in obesity and insulin resistance in a mouse model [140,141].

At the same time, a decrease in LON levels is associated with aging and chronic stress and contributes to the development of pathologies [142]. LON is involved in the regulation of hepatic insulin resistance and gluconeogenesis. Treatment with LON-specific miRNAs leads to mitochondrial dysfunction (decrease in ROS content, mitochondrial membrane potential). LON deficiency induces gluconeogenesis in the liver by inducing glucose-6-phosphatase and PGC-1a in human liver cells (SK-HEP-1). At the same time, overexpression of LON enhances hepatic insulin resistance in cells with dysfunctional mitochondria [143].

This suggests that mitochondrial proteases enhance systemic energy metabolism by regulating mitochondrial function and mitochondrial quality control [139].

A link between LONP1 and metabolic disorders has been demonstrated by the presence of low LONP1 expression in the liver of diabetic db/db mice [143] and by the fact that a reduction in LONP1 expression leads to impaired insulin signaling and increased expression of gluconeogenic enzymes in human liver cells [143]. It was found that the expression of LONP1 in visceral adipose tissue (VAT) was significantly higher in subjects with high body mass index than in subjects with low body mass index. In addition, high expression of LONP1 in the GTEx database was associated with increased lipid and glucose metabolism. These results suggest a modulatory effect of LONP1 VAT on systemic glucose metabolism in response to obesity. Glucose tolerance was associated with the expression of LONP1 in sWAT in BXD RI mice. However, no correlation was found between LONP1 VAT expression and serum glucose, lipid profiles, and HOMA-IR [137].

This suggests that a homeostatic mechanism to maintain mitochondrial function and systemic metabolism (including glucose) is initiated by increasing the expression of LONP1 in human VAT in obesity [137].

One study found global downregulation of mitochondrial oxidative pathways, mtDNA levels, and OXPHOS protein expression in subcutaneous adipose tissue of humans with high BMI compared with identical twins with lower body mass index [144]. However, the results of another study showed no suppression of gene expression [145]. When the mitochondrial function was analyzed, oxygen consumption rate and citrate synthase activity were significantly lower in obese human adipocytes, although mitochondrial content in adipocytes was not significantly different from that of the normal group [146].

Interestingly, the expression of LONP1 in muscle tissue serves as a mitochondrial sensor that responds to changes in the level of physical activity [147].

It was found that a twofold increase in the expression of LONP1 in the heart and a longer lifespan were observed in animals (mice) that were physically active throughout their lives compared to sedentary mice [134].

Hypoxia-induced factor 1 (HIF-1) regulates the composition of COX4 units [126]. At the same time, HIF-1 enhances Lon transcription and its overexpression contributes to COX4 degradation. Altering COX4 subunit expression allows the optimization of mitochondrial ATP production, oxygen consumption, and ROS formation [148].

The mitochondrial matrix is known to contain two highly conserved chaperone systems, HSP60 and mitochondrial HSP70 (mtHSP70), which are critical for facilitating the folding reaction of mitochondrial precursor proteins [149].

Interestingly, in silico analysis of the promoters of the *Hsp60*, *Hsp10*, and *Lonp1* genes revealed that these genes can be regulated by MAPK signaling [150], which has been linked to the development of obesity and MetS [151,152].

The chaperone activity of Lonp1 was also confirmed, reflecting the cooperation of Lonp1 with mtHSP70 in the biogenesis of mitochondrial matrix proteins [149].

Human Lon can bind to mitochondrial promoters and control mtDNA transcription/translation by degrading several regulatory proteins involved in mtDNA maintenance (TFAM, POLG) [153,154]. Enzymatically active murine Lon has been shown to hydrolyze ATP, cleave protein substrates in an ATP-induced manner, and bind specifically to single-stranded (not double-stranded) DNA [155]. Human Lon binds to mtDNA sequences containing at least four contiguous guanine residues capable of forming G-quadruplexes (quadruplex structures with tetrad arrangement of guanines) [156]. At the same time, suppression of Lon in human fibroblasts was associated with loss of mtDNA [157].

However, there are conflicting data showing no association between Lon suppression and mtDNA CN [158], which is contradicted by other studies [153,159].

Therefore, further studies to identify the molecular mechanisms of action of Lonp1 in different cell types/tissues in obesity/MetS and its role in regulating mtDNA will provide new perspectives and a basis for developing effective prognostic and therapeutic pathogenesis-based strategies for the treatment of metabolic diseases.

### 3.2. Nuclear Localization of Lonp1

A novel role for LonP1 has been identified in metabolic remodeling [127]. This suggests the possible existence of unexplored LonP1 functions [160].

Polo et al. identified mitochondria-associated membranes (MAMs) as a localization site for LonP1 in the presence of ER stress, suggesting that LonP1 is not exclusive to mitochondria [160]. These studies have been validated in other cell and animal models [161,162]. Lon proteases have also been identified in cellular organelles involved in cellular oxidative stress and oxygen utilization, the peroxisomes [163].

In addition, the movement of some mitochondrial proteins into the nucleus in response to various stimuli was previously uncovered, adding many additional functions (monooxygenase CLK-1 and transcription factor ATFS-1) to regulation. The nuclear and mitochondrial forms of Lonp1 were found to be identical, whereas the mitochondrial and nuclear forms of CLK-1 and ATFS-1 differed [164]. The greatest similarity is between Lonp1 and the mitochondrial pyruvate dehydrogenase complex (PDC), which moves from the mitochondrial matrix to the nucleus during the cell cycle in response to growth signals (serum, epidermal growth factor) [164].

Up to 22% of Lonp1 has been found to be localized in the nucleus. At the same time, their amount depends on the response of the cell to heat shock. In the nucleus, LonP1 interacts with heat shock factor 1 (HSF1) and modulates the heat shock response [164]. Work in prokaryotes confirms the complex interplay between LonP1 (known as La protease in *E. coli*) and heat shock [165]. Similar results were obtained when bacterial Lon was studied [166,167].

Lonp1 has been shown to be stress sensitive and activated upon heat shock in human cells [168]. Even heat shock exposure alone (no genotoxic stress, no cell cycle block) results in the translocation of Lonp1 to the nucleus [164]. Physical interaction of Lonp1 with HSF1 occurs in the nucleus, Lonp1-mediated modulation of HSF1 levels (by degradation of HSF1), and down-regulation of HSF1 target genes [164]. The manner in which Lonp1 is exported from mitochondria to the nucleus is still unknown, as is the nature of the interaction between Lonp1 and HSF1.

Thus, the AAA + Lonp1 protease has a number of functional properties that remain to be investigated. At the same time, special attention needs to be paid to investigating the possible therapeutic potential of Lonp1 in reducing the proinflammatory response and restoring mitochondrial dysfunction (mtDNA repair) in MetS and its components.

## 4. Conclusions

Metabolic syndrome is a collection of diseases that co-occur and increase the risk of developing cardiovascular disease [3]. The data presented above point to the important role of mitochondrial dysfunction in the development and progression of metabolic syndrome components, particularly disorders related to a key component of the mitochondrial genome—mtDNA: methylation, heteroplasmy, mtDNA copy number, and activation of signaling pathways associated with response to mtDNA as alarmins.

Despite the results obtained and new data in the field of the study of the components of metabolic syndrome and associated mitochondrial disorders, many fundamental questions have not yet been investigated and require more detailed analysis.

Studies conducted in larger populations in different countries will help to establish more precise molecular genetic links between mtDNA alterations and metabolic syndrome disorders and fill knowledge gaps regarding the pathogenesis of this disease. At the same time, studying the repair potential of cells, especially their organelles—mitochondria—will help develop a system for diagnosing metabolic disorders and identify targeted molecules for pathogenetically based treatment. One candidate for successful use in future developments may be the AAA + Lonp1 protease, given its broad spectrum of activity and function and its recently identified nuclear localization [164]. Further studies will allow us to determine the specific role of Lonp1 in the pathogenesis of each of the components of the metabolic syndrome (and their combination) and to develop drugs based on the Lonp1 protease, similar to the described new anticancer drugs targeting AAA + proteases [169].

In our opinion, molecular genetic research focusing on mtDNA biology and mtDNA repair processes will provide an effective personalized solution to prevent and combat the heterogeneous phenotypes of metabolic syndrome.

## Data Availability

No new data were created or analyzed in this study. Data sharing is not applicable to this article.

## References

[1] Révész D., Verhoeven J.E., Picard M., Lin J., Sidney S., Epel E.S., Penninx B.W.J.H., Puterman E. (2018). Associations Between Cellular Aging Markers and Metabolic Syndrome: Findings From the CARDIA Study. J. Clin. Endocrinol. Metab..

[2] Alkhulaifi F., Darkoh C. (2022). Meal Timing, Meal Frequency and Metabolic Syndrome. Nutrients.

[3] Saklayen M.G. (2018). The Global Epidemic of the Metabolic Syndrome. Curr. Hypertens. Rep..

[4] Reddy P., Lent-Schochet D., Ramakrishnan N., McLaughlin M., Jialal I. (2019). Metabolic Syndrome Is an Inflammatory Disorder: A Conspiracy between Adipose Tissue and Phagocytes. Clin. Chim. Acta.

[5] Ravaut G., Légiot A., Bergeron K.-F., Mounier C. (2020). Monounsaturated Fatty Acids in Obesity-Related Inflammation. Int. J. Mol. Sci..

[6] Mengel-From J., Thinggaard M., Dalgård C., Kyvik K.O., Christensen K., Christiansen L. (2014). Mitochondrial DNA Copy Number in Peripheral Blood Cells Declines with Age and Is Associated with General Health among Elderly. Hum. Genet..

[7] Zhang R., Wang Y., Ye K., Picard M., Gu Z. (2017). Independent Impacts of Aging on Mitochondrial DNA Quantity and Quality in Humans. BMC Genom..

[8] Gibellini L., De Gaetano A., Mandrioli M., Van Tongeren E., Bortolotti C.A., Cossarizza A., Pinti M. (2020). The Biology of Lonp1: More than a Mitochondrial Protease. Int. Rev. Cell Mol. Biol..

[9] Di Rienzo M., Romagnoli A., Ciccosanti F., Refolo G., Consalvi V., Arena G., Valente E.M., Piacentini M., Fimia G.M. (2022). AMBRA1 Regulates Mitophagy by Interacting with ATAD3A and Promoting PINK1 Stability. Autophagy.

[10] Zurita Rendón O., Shoubridge E.A. (2018). LONP1 Is Required for Maturation of a Subset of Mitochondrial Proteins, and Its Loss Elicits an Integrated Stress Response. Mol. Cell. Biol..

[11] Hosgood H.D., Liu C.-S., Rothman N., Weinstein S.J., Bonner M.R., Shen M., Lim U., Virtamo J., Cheng W., Albanes D. (2010). Mitochondrial DNA Copy Number and Lung Cancer Risk in a Prospective Cohort Study. Carcinogenesis.

[12] Riley J.S., Tait S.W. (2020). Mitochondrial DNA in Inflammation and Immunity. EMBO Rep..

[13] Dashti M., Alsaleh H., Eaaswarkhanth M., John S.E., Nizam R., Melhem M., Hebbar P., Sharma P., Al-Mulla F., Thanaraj T.A. (2021). Delineation of Mitochondrial DNA Variants From Exome Sequencing Data and Association of Haplogroups With Obesity in Kuwait. Front. Genet..

[14] Ding X., Fang T., Pang X., Pan X., Tong A., Lin Z., Zheng S., Zheng N. (2023). Mitochondrial DNA Abnormalities and Metabolic Syndrome. Front. Cell Dev. Biol..

[15] Viola A., Munari F., Sánchez-Rodríguez R., Scolaro T., Castegna A. (2019). The Metabolic Signature of Macrophage Responses. Front. Immunol..

[16] Castellani C.A., Longchamps R.J., Sun J., Guallar E., Arking D.E. (2020). Thinking Outside the Nucleus: Mitochondrial DNA Copy Number in Health and Disease. Mitochondrion.

[17] Guha M., Avadhani N.G. (2013). Mitochondrial Retrograde Signaling at the Crossroads of Tumor Bioenergetics, Genetics and Epigenetics. Mitochondrion.

[18] Jeng J.-Y., Yeh T.-S., Lee J.-W., Lin S.-H., Fong T.-H., Hsieh R.-H. (2008). Maintenance of Mitochondrial DNA Copy Number and Expression Are Essential for Preservation of Mitochondrial Function and Cell Growth. J. Cell. Biochem..

[19] Malik A.N., Czajka A. (2013). Is Mitochondrial DNA Content a Potential Biomarker of Mitochondrial Dysfunction?. Mitochondrion.

[20] Ren J., Pulakat L., Whaley-Connell A., Sowers J.R. (2010). Mitochondrial Biogenesis in the Metabolic Syndrome and Cardiovascular Disease. J. Mol. Med..

[21] Kim J.-A., Wei Y., Sowers J.R. (2008). Role of Mitochondrial Dysfunction in Insulin Resistance. Circ. Res..

[22] Liu J., Lloyd S.G. (2013). High-Fat, Low-Carbohydrate Diet Alters Myocardial Oxidative Stress and Impairs Recovery of Cardiac Function after Ischemia and Reperfusion in Obese Rats. Nutr. Res..

[23] Skuratovskaia D., Zatolokin P., Vulf M., Mazunin I., Litvinova L. (2019). Interrelation of Chemerin and TNF-α with MtDNA Copy Number in Adipose Tissues and Blood Cells in Obese Patients with and without Type 2 Diabetes. BMC Med. Genom..

[24] Meng S., Wu S., Liang L., Liang G., Giovannucci E., De Vivo I., Nan H. (2016). Leukocyte Mitochondrial DNA Copy Number, Anthropometric Indices, and Weight Change in US Women. Oncotarget.

[25] Skuratovskaia D.A., Sofronova J.K., Zatolokin P.A., Popadin K.Y., Vasilenko M.A., Litvinova L.S., Mazunin I.O. (2018). Additional Evidence of the Link between MtDNA Copy Number and the Body Mass Index. Mitochondrial DNA Part A DNA Mapp. Seq. Anal..

[26] Liu X., Longchamps R.J., Wiggins K.L., Raffield L.M., Bielak L.F., Zhao W., Pitsillides A., Blackwell T.W., Yao J., Guo X. (2021). Association of Mitochondrial DNA Copy Number with Cardiometabolic Diseases. Cell Genom..

[27] Koller A., Fazzini F., Lamina C., Rantner B., Kollerits B., Stadler M., Klein-Weigel P., Fraedrich G., Kronenberg F. (2020). Mitochondrial DNA Copy Number Is Associated with All-Cause Mortality and Cardiovascular Events in Patients with Peripheral Arterial Disease. J. Intern. Med..

[28] Agius R., Pace N.P., Fava S. (2022). Reduced Leukocyte Mitochondrial Copy Number in Metabolic Syndrome and Metabolically Healthy Obesity. Front. Endocrinol..

[29] Huang C.-H., Su S.-L., Hsieh M.-C., Cheng W.-L., Chang C.-C., Wu H.-L., Kuo C.-L., Lin T.-T., Liu C.-S. (2011). Depleted Leukocyte Mitochondrial DNA Copy Number in Metabolic Syndrome. J. Atheroscler. Thromb..

[30] Kim J.-H., Im J.-A., Lee D.-C. (2012). The Relationship between Leukocyte Mitochondrial DNA Contents and Metabolic Syndrome in Postmenopausal Women. Menopause.

[31] Fazzini F., Lamina C., Raftopoulou A., Koller A., Fuchsberger C., Pattaro C., Del Greco F.M., Döttelmayer P., Fendt L., Fritz J. (2021). Association of Mitochondrial DNA Copy Number with Metabolic Syndrome and Type 2 Diabetes in 14 176 Individuals. J. Intern. Med..

[32] Kim J.-H., Kim H.K., Ko J.-H., Bang H., Lee D.-C. (2013). The Relationship between Leukocyte Mitochondrial DNA Copy Number and Telomere Length in Community-Dwelling Elderly Women. PLoS ONE.

[33] Cree L.M., Patel S.K., Pyle A., Lynn S., Turnbull D.M., Chinnery P.F., Walker M. (2008). Age-Related Decline in Mitochondrial DNA Copy Number in Isolated Human Pancreatic Islets. Diabetologia.

[34] Picard M., Turnbull D.M. (2013). Linking the Metabolic State and Mitochondrial DNA in Chronic Disease, Health, and Aging. Diabetes.

[35] Memon A.A., Sundquist J., Hedelius A., Palmér K., Wang X., Sundquist K. (2021). Association of Mitochondrial DNA Copy Number with Prevalent and Incident Type 2 Diabetes in Women: A Population-Based Follow-up Study. Sci. Rep..

[36] Wang J., Liang H., Huang R., Weng X., Zheng L., Wang Y., Zheng X., Gu Z., Chen F., Shao J. (2023). Higher Mitochondrial DNA Copy Number Is Associated with Metformin-Induced Weight Loss. Commun. Med..

[37] Skuratovskaia D., Litvinova L., Vulf M., Zatolokin P., Popadin K., Mazunin I. (2019). From Normal to Obesity and Back: The Associations between Mitochondrial DNA Copy Number, Gender, and Body Mass Index. Cells.

[38] Matilainen O., Quirós P.M., Auwerx J. (2017). Mitochondria and Epigenetics—Crosstalk in Homeostasis and Stress. Trends Cell Biol..

[39] Castegna A., Iacobazzi V., Infantino V. (2015). The Mitochondrial Side of Epigenetics. Physiol. Genom..

[40] D’Aquila P., Bellizzi D., Passarino G. (2015). Mitochondria in Health, Aging and Diseases: The Epigenetic Perspective. Biogerontology.

[41] Castellani C.A., Longchamps R.J., Sumpter J.A., Newcomb C.E., Lane J.A., Grove M.L., Bressler J., Brody J.A., Floyd J.S., Bartz T.M. (2020). Mitochondrial DNA Copy Number Can Influence Mortality and Cardiovascular Disease via Methylation of Nuclear DNA CpGs. Genome Med..

[42] Murphy S.K., Huang Z., Hoyo C. (2012). Differentially Methylated Regions of Imprinted Genes in Prenatal, Perinatal and Postnatal Human Tissues. PLoS ONE.

[43] Fradin D., Boëlle P.-Y., Belot M.-P., Lachaux F., Tost J., Besse C., Deleuze J.-F., De Filippo G., Bougnères P. (2017). Genome-Wide Methylation Analysis Identifies Specific Epigenetic Marks In Severely Obese Children. Sci. Rep..

[44] Dou X., Boyd-Kirkup J.D., McDermott J., Zhang X., Li F., Rong B., Zhang R., Miao B., Chen P., Cheng H. (2019). The Stand-Biased Mitochondrial DNA Methylome And Its Regulation By DNMT3A. Genome Res..

[45] Guantes R., Rastrojo A., Neves R., Lima A., Aguado B., Iborra F.J. (2015). Global Variability in Gene Expression and Alternative Splicing Is Modulated by Mitochondrial Content. Genome Res..

[46] Bai Y., Guo Z., Xu J., Zhang J., Cui L., Zhang H., Zang S., Ai X. (2014). Single Nucleotide Polymorphisms In The D-Loop Region Of Mitochondrial DNA And Age-At0Onset Of Patients With Chronic Kidney Disease. Chin. Med. J..

[47] Stoccoro A., Smith A.R., Mosca L., Marocchi A., Gerardi F., Lunetta C., Cereda C., Gagliardi S., Lunnon K., Migliore L. (2020). Reduced Mitochondrial D-Loop Methylation Levels in Sporadic Amyotrophic Lateral Sclerosis. Clin. Epigenetics.

[48] Mposhi A., Van der Wijst M.G., Faber K.N., Rots M.G. (2017). Regulation of mitochondrial gene expression, the epigenetic enigma. Front. Biosci. (Landmark Ed).

[49] Dostal V., Churchill M.E.A. (2019). Cytosine Methylation of Mitochondrial DNA at CpG Sequences Impacts Transcription Factor A DNA Binding and Transcription. Biochim. Biophys. Acta Gene Regul. Mech..

[50] Gao J., Wen S., Zhou H., Feng S. (2015). De-Methylation of Displacement Loop of Mitochondrial DNA Is Associated with Increased Mitochondrial Copy Number and Nicotinamide Adenine Dinucleotide Subunit 2 Expression in Colorectal Cancer. Mol. Med. Rep..

[51] Park S.H., Lee S.Y., Kim S.A. (2021). Mitochondrial DNA Methylation Is Higher in Acute Coronary Syndrome Than in Stable Coronary Artery Disease. In Vivo.

[52] Low H.C., Chilian W.M., Ratnam W., Karupaiah T., Md Noh M.F., Mansor F., Ng Z.X., Pung Y.F. (2023). Changes in Mitochondrial Epigenome in Type 2 Diabetes Mellitus. Br. J. Biomed. Sci..

[53] Hao Z., Wu T., Cui X., Zhu P., Tan C., Dou X., Hsu K.W., Lin Y.T., Peng P.H., Zang L.S. (2020). N6-Deoxyadenosine Methylation In Mammalian Mitochondrial DNA. Mol. Cell.

[54] Bordoni L., Smerilli V., Nasuti C., Gabbianelli R. (2019). Mitochondrial DNA Methylation and Copy Number Predict Body Composition in a Young Female Population. J. Transl. Med..

[55] Bordoni L., Perugini J., Petracci I., Mercurio E.D., Lezoche G., Guerrieri M., Giordano A., Gabbianelli R. (2022). Mitochondrial DNA in Visceral Adipose Tissue in Severe Obesity: From Copy Number to D-Loop Methylation. Front. Biosci. (Landmark Ed).

[56] Corsi S., Iodice S., Vigna L., Cayir A., Mathers J.C., Bollati V., Byun H.-M. (2020). Platelet Mitochondrial DNA Methylation Predicts Future Cardiovascular Outcome in Adults with Overweight and Obesity. Clin. Epigenetics.

[57] Baccarelli A.A., Byun H.-M. (2015). Platelet Mitochondrial DNA Methylation: A Potential New Marker of Cardiovascular Disease. Clin. Epigenetics.

[58] Zheng L.D., Linarelli L.E., Liu L., Wall S.S., Greenawald M.H., Seidel R.W., Estabrooks P.A., Almeida F.A., Cheng Z. (2015). Insulin Resistance Is Associated with Epigenetic and Genetic Regulation of Mitochondrial DNA in Obese Humans. Clin. Epigenetics.

[59] Khotina V.A., Vinokurov A.Y., Bagheri Ekta M., Sukhorukov V.N., Orekhov A.N. (2023). Creation Of Mitochondrial Disease Models Using Mitochondrial DNA Editing. Biomrdicines.

[60] Katada S., Mito T., Ogasawara E., Hayashi J.-I., Nakada K. (2013). Mitochondrial DNA with a Large-Scale Deletion Causes Two Distinct Mitochondrial Disease Phenotypes in Mice. G3 (Bethesda).

[61] Farmer T., Naslavsky N., Caplan S. (2018). Tying Trafficking to Fusion and Fission at the Mighty Mitochondria. Traffic.

[62] Wallace D.C. (2005). A Mitochondrial Paradigm of Metabolic and Degenerative Diseases, Aging, and Cancer: A Dawn for Evolutionary Medicine. Annu. Rev. Genet..

[63] Palmieri V.O., De Rasmo D., Signorile A., Sardanelli A.M., Grattagliano I., Minerva F., Cardinale G., Portincasa P., Papa S., Palasciano G. (2011). T16189C Mitochondrial DNA Variant Is Associated with Metabolic Syndrome in Caucasian Subjects. Nutrition.

[64] Mueller E.E., Eder W., Ebner S., Schwaiger E., Santic D., Kreindl T., Stanger O., Paulweber B., Iglseder B., Oberkofler H. (2011). The Mitochondrial T16189C Polymorphism Is Associated with Coronary Artery Disease in Middle European Populations. PLoS ONE.

[65] Maassen J.A., ’T Hart L.M., Van Essen E., Heine R.J., Nijpels G., Jahangir Tafrechi R.S., Raap A.K., Janssen G.M.C., Lemkes H.H.P.J. (2004). Mitochondrial Diabetes: Molecular Mechanisms and Clinical Presentation. Diabetes.

[66] Park H., Davidson E., King M.P. (2003). The Pathogenic A3243G Mutation in Human Mitochondrial TRNALeu(UUR) Decreases the Efficiency of Aminoacylation. Biochemistry.

[67] Ye W., Chen S., Jin S., Lu J. (2013). A Novel Heteroplasmic Mitochondrial DNA Mutation, A8890G, in a Patient with Juvenile-onset Metabolic Syndrome: A Case Report. Mol. Med. Rep..

[68] Wallace D.C. (2015). Mitochondrial DNA Variation in Human Radiation and Disease. Cell.

[69] Ebner S., Mangge H., Langhof H., Halle M., Siegrist M., Aigner E., Paulmichl K., Paulweber B., Datz C., Sperl W. (2015). Mitochondrial Haplogroup T Is Associated with Obesity in Austrian Juveniles and Adults. PLoS ONE.

[70] Nardelli C., Labruna G., Liguori R., Mazzaccara C., Ferrigno M., Capobianco V., Pezzuti M., Castaldo G., Farinaro E., Contaldo F. (2013). Haplogroup T Is an Obesity Risk Factor: Mitochondrial DNA Haplotyping in a Morbid Obese Population from Southern Italy. Biomed. Res. Int..

[71] Dashti M., Alsaleh H., Rodriguez-Flores J.L., Eaaswarkhanth M., Al-Mulla F., Thanaraj T.A. (2021). Mitochondrial Haplogroup J Associated with Higher Risk of Obesity in the Qatari Population. Sci. Rep..

[72] Chalkia D., Chang Y.-C., Derbeneva O., Lvova M., Wang P., Mishmar D., Liu X., Singh L.N., Chuang L.-M., Wallace D.C. (2018). Mitochondrial DNA Associations with East Asian Metabolic Syndrome. Biochim. Biophys. Acta Bioenerg..

[73] Fuku N., Park K.S., Yamada Y., Nishigaki Y., Cho Y.M., Matsuo H., Segawa T., Watanabe S., Kato K., Yokoi K. (2007). Mitochondrial Haplogroup N9a Confers Resistance against Type 2 Diabetes in Asians. Am. J. Hum. Genet..

[74] Guo L.-J., Oshida Y., Fuku N., Takeyasu T., Fujita Y., Kurata M., Sato Y., Ito M., Tanaka M. (2005). Mitochondrial Genome Polymorphisms Associated with Type-2 Diabetes or Obesity. Mitochondrion.

[75] Fang H., Hu N., Zhao Q., Wang B., Zhou H., Fu Q., Shen L., Chen X., Shen F., Lyu J. (2018). MtDNA Haplogroup N9a Increases the Risk of Type 2 Diabetes by Altering Mitochondrial Function and Intracellular Mitochondrial Signals. Diabetes.

[76] Liao W.-Q., Pang Y., Yu C.-A., Wen J.-Y., Zhang Y.-G., Li X.-H. (2008). Novel Mutations of Mitochondrial DNA Associated with Type 2 Diabetes in Chinese Han Population. Tohoku J. Exp. Med..

[77] Niu Q., Zhang W., Wang H., Guan X., Lu J., Li W. (2015). Effects of Mitochondrial Haplogroup N9a on Type 2 Diabetes Mellitus and Its Associated Complications. Exp. Ther. Med..

[78] Loo J.-H., Trejaut J.A., Yen J.-C., Chen Z.-S., Ng W.-M., Huang C.-Y., Hsu K.-N., Hung K.-H., Hsiao Y., Wei Y.-H. (2014). Mitochondrial DNA Association Study of Type 2 Diabetes with or without Ischemic Stroke in Taiwan. BMC Res. Notes.

[79] Shen F.-C., Weng S.-W., Tsai M.-H., Su Y.-J., Li S.-C., Chang S.-J., Chen J.-F., Chang Y.-H., Liou C.-W., Lin T.-K. (2022). Mitochondrial Haplogroups Have a Better Correlation to Insulin Requirement than Nuclear Genetic Variants for Type 2 Diabetes Mellitus in Taiwanese Individuals. J. Diabetes Investig..

[80] Jiang W., Li R., Zhang Y., Wang P., Wu T., Lin J., Yu J., Gu M. (2017). Mitochondrial DNA Mutations Associated with Type 2 Diabetes Mellitus in Chinese Uyghur Population. Sci. Rep..

[81] De Gaetano A., Solodka K., Zanini G., Selleri V., Mattioli A.V., Nasi M., Pinti M. (2021). Molecular Mechanisms of MtDNA-Mediated Inflammation. Cells.

[82] Collins L.V., Hajizadeh S., Holme E., Jonsson I.-M., Tarkowski A. (2004). Endogenously Oxidized Mitochondrial DNA Induces in Vivo and in Vitro Inflammatory Responses. J. Leukoc. Biol..

[83] Shimada K., Crother T.R., Karlin J., Dagvadorj J., Chiba N., Chen S., Ramanujan V.K., Wolf A.J., Vergnes L., Ojcius D.M. (2012). Oxidized Mitochondrial DNA Activates the NLRP3 Inflammasome during Apoptosis. Immunity.

[84] Pazmandi K., Agod Z., Kumar B.V., Szabo A., Fekete T., Sogor V., Veres A., Boldogh I., Rajnavolgyi E., Lanyi A. (2014). Oxidative Modification Enhances the Immunostimulatory Effects of Extracellular Mitochondrial DNA on Plasmacytoid Dendritic Cells. Free. Radic. Biol. Med..

[85] Kunkel G.H., Chaturvedi P., Tyagi S.C. (2016). Mitochondrial Pathways to Cardiac Recovery: TFAM. Heart Fail Rev..

[86] Piantadosi C.A. (2020). Mitochondrial DNA, Oxidants, and Innate Immunity. Free. Radic. Biol. Med..

[87] Wang L., Zhang Q., Yuan K., Yuan J. (2021). MtDNA in the Pathogenesis of Cardiovascular Diseases. Dis. Markers.

[88] Krychtiuk K.A., Wurm R., Ruhittel S., Lenz M., Huber K., Wojta J., Heinz G., Hülsmann M., Speidl W.S. (2020). Release of Mitochondrial DNA Is Associated with Mortality in Severe Acute Heart Failure. Eur. Heart J. Acute Cardiovasc. Care.

[89] Bliksøen M., Mariero L.H., Torp M.K., Baysa A., Ytrehus K., Haugen F., Seljeflot I., Vaage J., Valen G., Stensløkken K.-O. (2016). Extracellular MtDNA Activates NF-ΚB via Toll-like Receptor 9 and Induces Cell Death in Cardiomyocytes. Basic Res. Cardiol..

[90] Donath M.Y., Meier D.T., Böni-Schnetzler M. (2019). Inflammation in the Pathophysiology and Therapy of Cardiometabolic Disease. Endocr. Rev..

[91] Pinti M., Cevenini E., Nasi M., De Biasi S., Salvioli S., Monti D., Benatti S., Gibellini L., Cotichini R., Stazi M.A. (2014). Circulating Mitochondrial DNA Increases with Age and Is a Familiar Trait: Implications for “Inflamm-Aging. ” Eur. J. Immunol..

[92] Ueda H., Yamaguchi O., Taneike M., Akazawa Y., Wada-Kobayashi H., Sugihara R., Yorifuji H., Nakayama H., Omiya S., Murakawa T. (2019). Administration of a TLR9 Inhibitor Attenuates the Development and Progression of Heart Failure in Mice. JACC Basic Transl. Sci..

[93] Liu Y., Jesus A.A., Marrero B., Yang D., Ramsey S.E., Sanchez G.A.M., Tenbrock K., Wittkowski H., Jones O.Y., Kuehn H.S. (2014). Activated STING in a Vascular and Pulmonary Syndrome. N. Engl. J. Med..

[94] West A.P., Khoury-Hanold W., Staron M., Tal M.C., Pineda C.M., Lang S.M., Bestwick M., Duguay B.A., Raimundo N., MacDuff D.A. (2015). Mitochondrial DNA Stress Primes the Antiviral Innate Immune Response. Nature.

[95] Sun L., Wu J., Du F., Chen X., Chen Z.J. (2013). Cyclic GMP-AMP Synthase Is a Cytosolic DNA Sensor That Activates the Type I Interferon Pathway. Science.

[96] Zhang C., Shang G., Gui X., Zhang X., Bai X.-C., Chen Z.J. (2019). Structural Basis of STING Binding with and Phosphorylation by TBK1. Nature.

[97] Abe T., Barber G.N. (2014). Cytosolic-DNA-Mediated, STING-Dependent Proinflammatory Gene Induction Necessitates Canonical NF-ΚB Activation through TBK1. J. Virol..

[98] Rodríguez-Nuevo A., Zorzano A. (2019). The Sensing of Mitochondrial DAMPs by Non-Immune Cells. Cell Stress.

[99] Liu S., Cai X., Wu J., Cong Q., Chen X., Li T., Du F., Ren J., Wu Y.-T., Grishin N.V. (2015). Phosphorylation of Innate Immune Adaptor Proteins MAVS, STING, and TRIF Induces IRF3 Activation. Science.

[100] Balka K.R., Louis C., Saunders T.L., Smith A.M., Calleja D.J., D’Silva D.B., Moghaddas F., Tailler M., Lawlor K.E., Zhan Y. (2020). TBK1 and IKKε Act Redundantly to Mediate STING-Induced NF-ΚB Responses in Myeloid Cells. Cell Rep..

[101] Hopfner K.-P., Hornung V. (2020). Molecular Mechanisms and Cellular Functions of CGAS-STING Signalling. Nat. Rev. Mol. Cell Biol..

[102] Oduro P.K., Zheng X., Wei J., Yang Y., Wang Y., Zhang H., Liu E., Gao X., Du M., Wang Q. (2022). The CGAS-STING Signaling in Cardiovascular and Metabolic Diseases: Future Novel Target Option for Pharmacotherapy. Acta Pharm. Sin. B.

[103] Yuzefovych L.V., Pastukh V.M., Ruchko M.V., Simmons J.D., Richards W.O., Rachek L.I. (2019). Plasma Mitochondrial DNA Is Elevated in Obese Type 2 Diabetes Mellitus Patients and Correlates Positively with Insulin Resistance. PLoS ONE.

[104] Nie S., Lu J., Wang L., Gao M. (2020). Pro-Inflammatory Role of Cell-Free Mitochondrial DNA in Cardiovascular Diseases. IUBMB Life.

[105] Andreeva L., Hiller B., Kostrewa D., Lässig C., de Oliveira Mann C.C., Jan Drexler D., Maiser A., Gaidt M., Leonhardt H., Hornung V. (2017). CGAS Senses Long and HMGB/TFAM-Bound U-Turn DNA by Forming Protein-DNA Ladders. Nature.

[106] Kumari M., Wang X., Lantier L., Lyubetskaya A., Eguchi J., Kang S., Tenen D., Roh H.C., Kong X., Kazak L. (2016). IRF3 Promotes Adipose Inflammation and Insulin Resistance and Represses Browning. J. Clin. Investig..

[107] Bai J., Cervantes C., Liu J., He S., Zhou H., Zhang B., Cai H., Yin D., Hu D., Li Z. (2017). DsbA-L Prevents Obesity-Induced Inflammation and Insulin Resistance by Suppressing the MtDNA Release-Activated CGAS-CGAMP-STING Pathway. Proc. Natl. Acad. Sci. USA.

[108] Zhou L., Liu M., Zhang J., Chen H., Dong L.Q., Liu F. (2010). DsbA-L Alleviates Endoplasmic Reticulum Stress-Induced Adiponectin Downregulation. Diabetes.

[109] Bai J., Cervantes C., He S., He J., Plasko G.R., Wen J., Li Z., Yin D., Zhang C., Liu M. (2020). Mitochondrial Stress-Activated CGAS-STING Pathway Inhibits Thermogenic Program and Contributes to Overnutrition-Induced Obesity in Mice. Commun. Biol..

[110] Zhao P., Wong K.I., Sun X., Reilly S.M., Uhm M., Liao Z., Skorobogatko Y., Saltiel A.R. (2018). TBK1 at the Crossroads of Inflammation and Energy Homeostasis in Adipose Tissue. Cell.

[111] Guo Z., Tang N., Liu F.-Y., Yang Z., Ma S.-Q., An P., Wu H.-M., Fan D., Tang Q.-Z. (2020). TLR9 Deficiency Alleviates Doxorubicin-Induced Cardiotoxicity via the Regulation of Autophagy. J. Cell. Mol. Med..

[112] Oka T., Hikoso S., Yamaguchi O., Taneike M., Takeda T., Tamai T., Oyabu J., Murakawa T., Nakayama H., Nishida K. (2012). Mitochondrial DNA That Escapes from Autophagy Causes Inflammation and Heart Failure. Nature.

[113] Xie L., He S., Kong N., Zhu Y., Tang Y., Li J., Liu Z., Liu J., Gong J. (2018). Cpg-ODN, a TLR9 Agonist, Aggravates Myocardial Ischemia/Reperfusion Injury by Activation of TLR9-P38 MAPK Signaling. Cell. Physiol. Biochem..

[114] Pelka K., Phulphagar K., Zimmermann J., Stahl R., Schmid-Burgk J.L., Schmidt T., Spille J.-H., Labzin L.I., Agrawal S., Kandimalla E.R. (2014). Cutting Edge: The UNC93B1 Tyrosine-Based Motif Regulates Trafficking and TLR Responses via Separate Mechanisms. J. Immunol..

[115] Devaraj S., Adams-Huet B., Jialal I. (2015). Endosomal Toll-Like Receptor Status in Patients with Metabolic Syndrome. Metab. Syndr. Relat. Disord..

[116] Nishimoto S., Fukuda D., Higashikuni Y., Tanaka K., Hirata Y., Murata C., Kim-Kaneyama J.R., Sato F., Bando M., Yagi S. (2016). Obesity-induced DNA released from adipocytes stimulates chronic adipose tissue inflammation and insulin resistance. Sci. Adv..

[117] Ye W., Wen C., Zeng A., Hu X. (2023). Increased Levels of Circulating Oxidized Mitochondrial DNA Contribute to Chronic Inflammation in Metabolic Syndrome, and MitoQ-Based Antioxidant Therapy Alleviates This DNA-Induced Inflammation. Mol. Cell. Endocrinol..

[118] Broz P., Dixit V.M. (2016). Inflammasomes: Mechanism of Assembly, Regulation and Signalling. Nat. Rev. Immunol..

[119] Qiu Z., He Y., Ming H., Lei S., Leng Y., Xia Z.-Y. (2019). Lipopolysaccharide (LPS) Aggravates High Glucose- and Hypoxia/Reoxygenation-Induced Injury through Activating ROS-Dependent NLRP3 Inflammasome-Mediated Pyroptosis in H9C2 Cardiomyocytes. J. Diabetes Res..

[120] Pahwa R., Singh A., Adams-Huet B., Devaraj S., Jialal I. (2021). Increased Inflammasome Activity in Subcutaneous Adipose Tissue of Patients with Metabolic Syndrome. Diabetes/Metabolism Res. Rev..

[121] Vandanmagsar B., Youm Y.-H., Ravussin A., Galgani J.E., Stadler K., Mynatt R.L., Ravussin E., Stephens J.M., Dixit V.D. (2011). The NLRP3 Inflammasome Instigates Obesity-Induced Inflammation and Insulin Resistance. Nat. Med..

[122] Nakahira K., Haspel J.A., Rathinam V.A.K., Lee S.-J., Dolinay T., Lam H.C., Englert J.A., Rabinovitch M., Cernadas M., Kim H.P. (2011). Autophagy Proteins Regulate Innate Immune Responses by Inhibiting the Release of Mitochondrial DNA Mediated by the NALP3 Inflammasome. Nat. Immunol..

[123] Ward G.A., McGraw K.L., Abbas-Aghababazadeh F., Meyer B.S., McLemore A.F., Vincelette N.D., Lam N.B., Aldrich A.L., Al Ali N.H., Padron E. (2021). Oxidized Mitochondrial DNA Released after Inflammasome Activation Is a Disease Biomarker for Myelodysplastic Syndromes. Blood Adv..

[124] Crewe C., Schafer C., Lee I., Kinter M., Szweda L.I. (2017). Regulation of Pyruvate Dehydrogenase Kinase 4 in the Heart through Degradation by the Lon Protease in Response to Mitochondrial Substrate Availability. J. Biol. Chem..

[125] Sepuri N.B.V., Angireddy R., Srinivasan S., Guha M., Spear J., Lu B., Anandatheerthavarada H.K., Suzuki C.K., Avadhani N.G. (2017). Mitochondrial LON Protease-Dependent Degradation of Cytochrome c Oxidase Subunits under Hypoxia and Myocardial Ischemia. Biochim. Biophys. Acta Bioenerg..

[126] Bota D.A., Davies K.J.A. (2016). Mitochondrial Lon Protease in Human Disease and Aging: Including an Etiologic Classification of Lon-Related Diseases and Disorders. Free. Radic. Biol. Med..

[127] Quirós P.M., Español Y., Acín-Pérez R., Rodríguez F., Bárcena C., Watanabe K., Calvo E., Loureiro M., Fernández-García M.S., Fueyo A. (2014). ATP-Dependent Lon Protease Controls Tumor Bioenergetics by Reprogramming Mitochondrial Activity. Cell Rep..

[128] Kuo C.-Y., Chiu Y.-C., Lee A.Y.-L., Hwang T.-L. (2015). Mitochondrial Lon Protease Controls ROS-Dependent Apoptosis in Cardiomyocyte under Hypoxia. Mitochondrion.

[129] Nie X., Li M., Lu B., Zhang Y., Lan L., Chen L., Lu J. (2013). Down-Regulating Overexpressed Human Lon in Cervical Cancer Suppresses Cell Proliferation and Bioenergetics. PLoS ONE.

[130] Strauss K.A., Jinks R.N., Puffenberger E.G., Venkatesh S., Singh K., Cheng I., Mikita N., Thilagavathi J., Lee J., Sarafianos S. (2015). CODAS Syndrome Is Associated with Mutations of LONP1, Encoding Mitochondrial AAA+ Lon Protease. Am. J. Hum. Genet..

[131] Dikoglu E., Alfaiz A., Gorna M., Bertola D., Chae J.H., Cho T.-J., Derbent M., Alanay Y., Guran T., Kim O.-H. (2015). Mutations in LONP1, a Mitochondrial Matrix Protease, Cause CODAS Syndrome. Am. J. Med. Genet. A.

[132] Venkatesh S., Li M., Saito T., Tong M., Rashed E., Mareedu S., Zhai P., Bárcena C., López-Otín C., Yehia G. (2019). Mitochondrial LonP1 Protects Cardiomyocytes from Ischemia/Reperfusion Injury in Vivo. J. Mol. Cell. Cardiol..

[133] Mottis A., Jovaisaite V., Auwerx J. (2014). The Mitochondrial Unfolded Protein Response in Mammalian Physiology. Mamm. Genome.

[134] Welle S., Glueck S.B. (2003). In for the Long Run: Focus on “Lifelong Voluntary Exercise in the Mouse Prevents Age-Related Alterations in Gene Expression in the Heart. ” Physiol. Genom..

[135] Jovaisaite V., Mouchiroud L., Auwerx J. (2014). The Mitochondrial Unfolded Protein Response, a Conserved Stress Response Pathway with Implications in Health and Disease. J. Exp. Biol..

[136] Held N.M., Houtkooper R.H. (2015). Mitochondrial Quality Control Pathways as Determinants of Metabolic Health. Bioessays.

[137] Lee J.H., Jung S.-B., Lee S.E., Kim J.E., Kim J.T., Kang Y.E., Kang S.G., Yi H.-S., Ko Y.B., Lee K.H. (2021). Expression of LONP1 Is High in Visceral Adipose Tissue in Obesity, and Is Associated with Glucose and Lipid Metabolism. Endocrinol. Metab..

[138] Deshwal S., Fiedler K.U., Langer T. (2020). Mitochondrial Proteases: Multifaceted Regulators of Mitochondrial Plasticity. Annu. Rev. Biochem..

[139] Yi H.-S., Chang J.Y., Shong M. (2018). The Mitochondrial Unfolded Protein Response and Mitohormesis: A Perspective on Metabolic Diseases. J. Mol. Endocrinol..

[140] Choi M.J., Jung S.-B., Lee S.E., Kang S.G., Lee J.H., Ryu M.J., Chung H.K., Chang J.Y., Kim Y.K., Hong H.J. (2020). An Adipocyte-Specific Defect in Oxidative Phosphorylation Increases Systemic Energy Expenditure and Protects against Diet-Induced Obesity in Mouse Models. Diabetologia.

[141] Chung H.K., Ryu D., Kim K.S., Chang J.Y., Kim Y.K., Yi H.-S., Kang S.G., Choi M.J., Lee S.E., Jung S.-B. (2017). Growth Differentiation Factor 15 Is a Myomitokine Governing Systemic Energy Homeostasis. J. Cell Biol..

[142] Rigotto G., Basso E. (2019). Mitochondrial Dysfunctions: A Thread Sewing Together Alzheimer’s Disease, Diabetes, and Obesity. Oxid. Med. Cell. Longev..

[143] Lee H.J., Chung K., Lee H., Lee K., Lim J.H., Song J. (2011). Downregulation of Mitochondrial Lon Protease Impairs Mitochondrial Function and Causes Hepatic Insulin Resistance in Human Liver SK-HEP-1 Cells. Diabetologia.

[144] Heinonen S., Buzkova J., Muniandy M., Kaksonen R., Ollikainen M., Ismail K., Hakkarainen A., Lundbom J., Lundbom N., Vuolteenaho K. (2015). Impaired Mitochondrial Biogenesis in Adipose Tissue in Acquired Obesity. Diabetes.

[145] Dahlman I., Forsgren M., Sjögren A., Nordström E.A., Kaaman M., Näslund E., Attersand A., Arner P. (2006). Downregulation of Electron Transport Chain Genes in Visceral Adipose Tissue in Type 2 Diabetes Independent of Obesity and Possibly Involving Tumor Necrosis Factor-Alpha. Diabetes.

[146] Yin X., Lanza I.R., Swain J.M., Sarr M.G., Nair K.S., Jensen M.D. (2014). Adipocyte Mitochondrial Function Is Reduced in Human Obesity Independent of Fat Cell Size. J. Clin. Endocrinol. Metab..

[147] Xu Z., Fu T., Guo Q., Zhou D., Sun W., Zhou Z., Chen X., Zhang J., Liu L., Xiao L. (2022). Disuse-Associated Loss of the Protease LONP1 in Muscle Impairs Mitochondrial Function and Causes Reduced Skeletal Muscle Mass and Strength. Nat. Commun..

[148] Fukuda R., Zhang H., Kim J., Shimoda L., Dang C.V., Semenza G.L. (2007). HIF-1 Regulates Cytochrome Oxidase Subunits to Optimize Efficiency of Respiration in Hypoxic Cells. Cell.

[149] Shin C.-S., Meng S., Garbis S.D., Moradian A., Taylor R.W., Sweredoski M.J., Lomenick B., Chan D.C. (2021). LONP1 and MtHSP70 Cooperate to Promote Mitochondrial Protein Folding. Nat. Commun..

[150] Grzybek M., Palladini A., Alexaki V.I., Surma M.A., Simons K., Chavakis T., Klose C., Coskun Ü. (2019). Comprehensive and Quantitative Analysis of White and Brown Adipose Tissue by Shotgun Lipidomics. Mol. Metab..

[151] Donohoe F., Wilkinson M., Baxter E., Brennan D.J. (2020). Mitogen-Activated Protein Kinase (MAPK) and Obesity-Related Cancer. Int. J. Mol. Sci..

[152] Och A., Och M., Nowak R., Podgórska D., Podgórski R. (2022). Berberine, a Herbal Metabolite in the Metabolic Syndrome: The Risk Factors, Course, and Consequences of the Disease. Molecules.

[153] Matsushima Y., Goto Y.-I., Kaguni L.S. (2010). Mitochondrial Lon Protease Regulates Mitochondrial DNA Copy Number and Transcription by Selective Degradation of Mitochondrial Transcription Factor A (TFAM). Proc. Natl. Acad. Sci. USA.

[154] Fu G.K., Markovitz D.M. (1998). The Human LON Protease Binds to Mitochondrial Promoters in a Single-Stranded, Site-Specific, Strand-Specific Manner. Biochemistry.

[155] Lu B., Liu T., Crosby J.A., Thomas-Wohlever J., Lee I., Suzuki C.K. (2003). The ATP-Dependent Lon Protease of Mus Musculus Is a DNA-Binding Protein That Is Functionally Conserved between Yeast and Mammals. Gene.

[156] Chen S.-H., Suzuki C.K., Wu S.-H. (2008). Thermodynamic Characterization of Specific Interactions between the Human Lon Protease and G-Quartet DNA. Nucleic Acids Res..

[157] Bota D.A., Ngo J.K., Davies K.J.A. (2005). Downregulation of the Human Lon Protease Impairs Mitochondrial Structure and Function and Causes Cell Death. Free. Radic. Biol. Med..

[158] Lu B., Yadav S., Shah P.G., Liu T., Tian B., Pukszta S., Villaluna N., Kutejová E., Newlon C.S., Santos J.H. (2007). Roles for the Human ATP-Dependent Lon Protease in Mitochondrial DNA Maintenance. J. Biol. Chem..

[159] De Gaetano A., Gibellini L., Bianchini E., Borella R., De Biasi S., Nasi M., Boraldi F., Cossarizza A., Pinti M. (2020). Impaired Mitochondrial Morphology and Functionality in Lonp1wt/- Mice. J. Clin. Med..

[160] Polo M., Alegre F., Moragrega A.B., Gibellini L., Marti-Rodrigo A., Blas-Garcia A., Esplugues J.V., Apostolova N. (2017). Lon Protease: A Novel Mitochondrial Matrix Protein in the Interconnection between Drug-Induced Mitochondrial Dysfunction and Endoplasmic Reticulum Stress. Br. J. Pharmacol..

[161] Horner S.M., Wilkins C., Badil S., Iskarpatyoti J., Gale M. (2015). Proteomic Analysis of Mitochondrial-Associated ER Membranes (MAM) during RNA Virus Infection Reveals Dynamic Changes in Protein and Organelle Trafficking. PLoS ONE.

[162] Poston C.N., Krishnan S.C., Bazemore-Walker C.R. (2013). In-Depth Proteomic Analysis of Mammalian Mitochondria-Associated Membranes (MAM). J. Proteom..

[163] Pomatto L.C.D., Raynes R., Davies K.J.A. (2017). The Peroxisomal Lon Protease LonP2 in Aging and Disease: Functions and Comparisons with Mitochondrial Lon Protease LonP1. Biol. Rev..

[164] Gibellini L., Borella R., De Gaetano A., Zanini G., Tartaro D.L., Carnevale G., Beretti F., Losi L., De Biasi S., Nasi M. (2022). Evidence for Mitochondrial Lonp1 Expression in the Nucleus. Sci. Rep..

[165] Pinti M., Gibellini L., Liu Y., Xu S., Lu B., Cossarizza A. (2015). Mitochondrial Lon Protease at the Crossroads of Oxidative Stress, Ageing and Cancer. Cell. Mol. Life Sci..

[166] Bhattacharjee S., Dasgupta R., Bagchi A. (2017). Elucidation of the Molecular Mechanism of Heat Shock Proteins and Its Correlation with K722Q Mutations in Lon Protease. Biosystems.

[167] Puri N., Karzai A.W. (2017). HspQ Functions as a Unique Specificity-Enhancing Factor for the AAA+ Lon Protease. Mol. Cell.

[168] Ngo J.K., Davies K.J.A. (2009). Mitochondrial Lon Protease Is a Human Stress Protein. Free. Radic. Biol. Med..

[169] Wong K.S., Houry W.A. (2019). Recent Advances in Targeting Human Mitochondrial AAA+ Proteases to Develop Novel Cancer Therapeutics. Adv. Exp. Med. Biol..

